# Estrogen-Like Effects of Cadmium *in Vivo* Do Not Appear to be Mediated via the Classical Estrogen Receptor Transcriptional Pathway

**DOI:** 10.1289/ehp.1001967

**Published:** 2010-06-04

**Authors:** Imran Ali, Pauliina E. Penttinen-Damdimopoulou, Sari I. Mäkelä, Marika Berglund, Ulla Stenius, Agneta Åkesson, Helen Håkansson, Krister Halldin

**Affiliations:** 1 Institute of Environmental Medicine, Karolinska Institutet, Stockholm, Sweden; 2 Functional Foods Forum and Institute of Biomedicine, University of Turku, Turku, Finland

**Keywords:** cadmium, endocrine disruption, estrogen-like effects, metalloestrogens, estrogen receptors, uterus

## Abstract

**Background:**

Cadmium (Cd), a ubiquitous food contaminant, has been proposed to be an endocrine disruptor by inducing estrogenic responses *in vivo*. Several *in vitro* studies suggested that these effects are mediated via estrogen receptors (ERs).

**Objective:**

We performed this study to clarify whether Cd-induced effects *in vivo* are mediated via classical ER signaling through estrogen responsive element (ERE)-regulated genes or if other signaling pathways are involved.

**Methods:**

We investigated the estrogenic effects of cadmium chloride (CdCl_2_) exposure *in vivo* by applying the Organisation for Economic Co-operation and Development (OECD) rodent uterotrophic bioassay to transgenic ERE-luciferase reporter mice. Immature female mice were injected subcutaneously with CdCl_2_ (5, 50, or 500 μg/kg body weight) or with 17α-ethinylestradiol (EE_2_) on 3 consecutive days. We examined uterine weight and histology, vaginal opening, body and organ weights, Cd tissue retention, activation of mitogen-activated protein kinase (MAPK) pathways, and ERE-dependent luciferase expression.

**Results:**

CdCl_2_ increased the height of the uterine luminal epithelium in a dose-dependent manner without increasing the uterine wet weight, altering the timing of vaginal opening, or affecting the luciferase activity in reproductive or nonreproductive organs. However, we observed changes in the phosphorylation of mouse double minute 2 oncoprotein (Mdm2) and extracellular signal-regulated kinase (Erk1/2) in the liver after CdCl_2_ exposure. As we expected, EE_2_ advanced vaginal opening and increased uterine epithelial height, uterine wet weight, and luciferase activity in various tissues.

**Conclusion:**

Our data suggest that Cd exposure induces a limited spectrum of estrogenic responses *in vivo* and that, in certain targets, effects of Cd might not be mediated via classical ER signaling through ERE-regulated genes.

Cadmium (Cd) is a widespread nephrotoxic food contaminant that accumulates and has a particularly long biological half-life in the liver and kidney (Amzal et al. 2009; Järup and Åkesson 2009). More recently, Cd was proposed to possess endocrine-disruptive properties. Several well-characterized estrogenic responses were induced in rodents after intraperitoneal injection of low doses of Cd (Johnson et al. 2003). Effects included increased uterine weight, luminal epithelium height, hyperplasia and hypertrophy of the endometrial lining, induction of uterine progesterone receptor expression, and enhanced formation of side branches, and alveolar buds in the mammary gland. Many of the effects have been confirmed by other groups, however with higher doses (Alonso-Gonzalez et al. 2007; Höfer et al. 2009; Zhang et al. 2007).

Estrogen, because of its proliferative effects, is a key factor in the etiology of breast and endometrial cancer (Persson 2000). Consequently, pollutants that mimic the effects of estrogen have been suggested to contribute to the high incidence of hormone-related cancers in Western populations (Davis et al. 1993; Safe 2004). The observed associations between long-term dietary Cd exposure and endometrial cancer incidence (Åkesson et al. 2008) and between urinary Cd and risk of breast cancer (McElroy et al. 2006) support this hypothesis. However, little is known about the possible mechanisms of action involved in the estrogenic responses associated with Cd exposure.

The classical mode of action of estrogens is mediated by transcriptional actions of the nuclear estrogen receptors, ERα and ERβ. In the classical estrogen signaling pathway, these receptors regulate transcription through direct interaction with specific DNA binding sites—estrogen-responsive elements (EREs)—present in the promoter regions of target genes (Edwards 2005; Nilsson et al. 2001). Estrogens can also signal through a nonclassical genomic pathway, where the ERs modulate transcription by tethering to other transcription factors, such as Sp1 and activator protein-1 (AP-1), instead of binding directly to DNA. The rapid biochemical and physiological effects of estrogens indicate that an alternative pathway, acting at the level of the plasma membrane (plasma membrane–associated signaling) should also be considered. In this process, various cytosolic kinase signaling pathways are activated (Acconcia and Kumar 2006; Fu and Simoncini 2008; Levin and Pietras 2008).

Several *in vitro* studies have shown that Cd can activate ERα, increase ERα-mediated cell proliferation, induce mitogenesis, and have mutagenic properties (Brama et al. 2007; Jin et al. 2003; Misra et al. 2002; Siewit et al. 2010; Slebos et al. 2006, Stoica et al. 2000). In a recent study, Benbrahim-Tallaa et al. (2009) also showed that Cd malignantly transforms normal ER-negative human breast epithelial cells. Furthermore, in breast cancer cell lines, Cd has been shown to cause rapid activation of extracellular signal-regulated kinase (Erk)1/2 and Akt, suggesting membrane-associated estrogen signaling (Liu et al. 2008). In contrast, some *in vitro* estrogenicity assays showed no Cd-induced activity (Le Guevel et al. 2000; Silva et al. 2006; Vetillard and Bailhache 2005).

Because of the inconsistent and inconclusive findings on the estrogenicity of Cd both *in vitro* and *in vivo* and because of the possible link to the risk of hormonal cancers in humans, we investigated the estrogenic responses of Cd in mice using a slightly modified version of the rodent uterotrophic bioassay [Organisation for Economic Co-operation and Development (OECD) 2007]. This bioassay is a widely used *in vivo* system used to detect the estrogenic properties of chemicals and has been applied in many studies. To assess the tissue-specific activation of classical genomic estrogen signaling, we exposed transgenic immature female ERE-luciferase mice (Lemmen et al. 2004) to cadmium chloride (CdCl_2_) or ethinylestradiol (EE_2_). In addition, we assessed the activation of mitogen-activated protein kinase (MAPK) pathways. Our main objectives were to provide new knowledge on the mechanisms involved in the estrogenic effects of Cd *in vivo* and to examine the sensitivity of these effects at Cd exposure levels of relevance for health risk assessment considerations.

## Materials and Methods

### Animals

In this study we used immature mice carrying an estrogen-responsive reporter gene construct (3xERE-TATA-luciferase) consisting of trimerized ERE coupled to a minimal TATA-box driving the expression of luciferase, described in detail by Legler et al. (1999) and Lemmen et al. (2004). The animals in our study were derived from the β-globin insulated ERE-luc line INS7 (Lemmen et al. 2004). The animal experiments were performed at the animal department of the University of Turku under license numbers 1592/05 and ESLH-2007-03984. Animals used in this study were treated humanely and with regard for alleviation of suffering. All experimental animals were kept under standard housing conditions on a 12:12-hr light/dark cycle at 22 ± 1°C and 50–60% relative humidity. Animals had free access to purified AIN-93G rodent diet [Special Diet Services (SDS), Whitham, Essex, UK] (Reeves et al. 1993) with slight modifications: Soy oil was replaced with rape seed oil to eliminate all possible residual phytoestrogens from the diet (Penttinen-Damdimopoulou et al. 2009).

### Subcutaneous injection of CdCl_2_ and EE_2_

Nineteen-day-old female mice weighing 7–9 g, randomly selected for each treatment group, received CdCl_2_ (Sigma, St. Louis, MO, USA) dissolved in sterile phosphate-buffered saline (PBS) at 5 (*n =* 6), 50 (*n =* 6), and 500 μg/kg body weight (BW) (*n =* 5) per day for 3 consecutive days via subcutaneous (SC) injections. Control animals (*n =* 5) were injected with sterile PBS. We used EE_2_ (Sigma) dissolved in corn oil (50 μg/kg BW, SC) as a positive control (*n =* 5).

### Tissue collection

One day after the last injection, (the fourth day of the experiment), the 22-day-old mice were examined for vaginal opening, euthanized by carbon dioxide asphyxiation, and weighed. Subsequently, whole blood was drawn by heart puncture, and tissues [brain, pituitary gland, mammary gland, white adipose tissue (WAT), ovary, uterus, vagina, liver, kidney, heart, and lungs] were collected into microcentrifuge tubes, snap-frozen in liquid nitrogen, and stored at −80°C until analysis. We recorded organ weights for uterus, liver, and kidney. For Cd analysis, one kidney and a piece of liver were placed separately into sterile acid-washed microcentrifuge tubes (StarLab, Ahrensburg, Germany) and kept on dry ice before storage at −80°C. One horn of the uterus was fixed in 10% formalin (Oy FF-Chemicals AB, Haukipudas, Finland) overnight and preserved in 70% ethanol (Kemetyl AB, Huddinge, Sweden) for histological evaluation.

### Cd analysis

We analyzed Cd concentration in liver and kidney, the main organs of accumulation, using inductively coupled plasma mass spectrometry (Agilent 7500ce; Agilent Technologies, Waldbronn, Germany) with a collision/reaction cell system autosampler (Cetac ASX-510; Cetac Technologies, Omaha, NE, USA), an integrated sample introduction system, and a MicroMist nebulizer (Agilent Technologies, Waldbronn, Germany) in quartz (Kippler et al. 2009). Digestion of tissue samples was performed with a Milestone ultraCLAVE II microwave digestion system (EMLS, Leutkirch, Germany). Approximately 0.5 g sample was mixed with 2 mL nitric acid (65% suprapur; Merck, Darmstadt, Germany) and 3 mL deionized water, and then digested at 250°C for 30 min in the autoclave. For quality control, we analyzed the following reference samples: NBS bovine liver 1577 (National Bureau of Standards, Washington, DC, USA), CRM 185 bovine liver, CRM 186 pig kidney, Seronorm MR4206 blood (BCR, Brussels, Belgium), and Seronorm 0503109 blood (SERO AS, Billingstad, Norway); results for all reference samples were at their target value. The limit of detection (LOD) was 0.004 ng/g, calculated as mean of digested blanks + 3 SDs. All the measured concentrations were above the calculated LOD.

### Leuciferas activity measurements

We measured luciferase activity in individual samples as previously described by Penttinen et al. (2007). Relative light units (RLUs) were calculated as


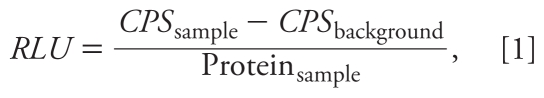


where *CPS* is counts per second, *CPS*_background_ and *CPS*_sample_ are the average CPS values measured from each plate before and after the luciferase reaction, respectively, and Protein_sample_ refers to the protein concentration measured in each sample.

Briefly, frozen liver, kidney, lungs, brain, heart, mammary gland, and WAT samples were minced by hammering on a cold metal surface, and immediately transferred to 12-mL tubes together with lysis buffer [25 mM Tris-acetate (pH 7.8), 1.5 mM EDTA, 10% glycerol, 1% Triton-X, 2 mM dithiothreitol (Sigma) containing Complete Mini-Protease Inhibitor Tablets (Roche Diagnostics GmbH, Penzberg, Germany)]. Pituitary gland, uterus, vagina, and ovary tissue samples were homogenized directly in lysis buffer with a micropestle. Homogenates were centrifuged at 4°C, 4,000 rpm, for 30 min and supernatants were collected into sterile microcentrifuge tubes. Luciferase activity was measured using the BioThema Luciferase Assay Kit (BioThema, Haninge, Sweden) in 96-well plates (Greiner, Frickenhausen, Germany). Luminescence was recorded with a Victor^2^ 1420 Multilabel analyzer (PerkinElmer, Turku, Finland), and protein contents were measured using Bradford assay reagents (Bio-Rad Protein Assay; Bio-Rad, Hercules, CA, USA) on a Shimadzu UV-1601 spectrophotometer (Shimadzu, Kyoto, Japan). Concentrations were determined by comparison with bovine serum albumin (BSA) standards (BSA Fraction V; Sigma).

### Western blot analysis of pErk, pAkt, and phospho-mouse double minute 2 oncoprotein (pMdm2) in liver

We quantified the protein contents in frozen liver homogenates using Coomassie Plus Protein Assay Reagent (Pierce, Täby, Sweden). The samples were subjected to SDS-PAGE, and the separated proteins were transferred to a polyvinylidene difluoride membrane (Bio-Rad) and subsequently probed with primary antibodies {pMdm2 (Ser166) from Cell Signaling Technology (Danvers, MA, USA); and phospho-Erk [Tyr204 (E-4), sc-7383], phospho-Akt (Ser473) sc-7985, and cyclin-dependent kinase 2 [Cdk2 (M2), sc-163] from Santa Cruz Biotechnology (Santa Cruz, CA, USA)} and then secondary antibodies [goat anti-rabbit IgG (sc-2004) and goat anti-mouse IgG (sc-2005) from Santa Cruz Biotechnology]. Cdk2 was used as a loading control. The results were visualized using the ECL detection kit (Amersham GE Healthcare Bio-sciences AB, Uppsala, Sweden). The densitometric analysis was performed using ImageJ version 1.41o software (National Institutes of Health, Bethesda, MD, USA; http://rsbweb.nih.gov/ij/index.html).

### Histological evaluation of the uterus

Sections of paraffin-embedded uterine tissues (6 μm) were mounted on SuperFrost Plus slides (Menzel-Gläzer, Braunschweig, Germany) followed by clearing, rehydration, and staining with hematoxylin and eosin (Fischer et al. 2006). All sections (five or six per animal) were examined under a Diaplan light microscope (Leitz, Wetzlar, Germany), and photos were taken for computerized image analysis. The height of luminal epithelium (micrometers) was measured at 10 different locations in two sections per animal to get an average value of luminal epithelium height.

### Statistical analysis

Unless stated otherwise, all reported values are expressed as mean ± SD. For luciferase measurements, data are expressed as median and range. We used one-way analysis of variance to compare the effect among different treatment groups. For values that were statistically significant, we applied Dunnett’s multiple comparison test to make post hoc comparisons between the means of control and treatment groups. Luciferase activity data were log transformed before statistical analysis. Differences were considered significant at *p* < 0.05 for two-tailed tests.

## Results

### Vaginal opening and body, liver, and kidney weights

To examine the onset of puberty, we monitored the vaginal opening after EE_2_ and CdCl_2_ treatments. All EE_2_-treated animals displayed vaginal opening on the last day of the experiment (22 days of age), indicating advanced onset of puberty. On the same day, no vaginal opening was observed in any of the CdCl_2_-treated or PBS control animals.

To investigate the signs of general toxicity after CdCl_2_ exposure, we measured body weights and relative liver and kidney weights. These weights were not altered in the CdCl_2_- or EE_2_-treated animals compared with PBS controls (Table 1), with one exception: EE_2_ caused an increase in relative liver weight.

### Cd concentrations in liver and kidney

**Figure 1:**
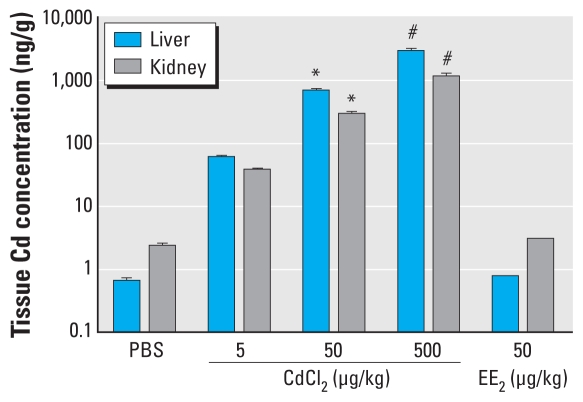
Tissue Cd concentration (mean ± SD) in the liver and kidney after SC exposure to CdCl_2_, EE_2_, or PBS for 3 consecutive days. *p < 0.05, and ^#^*p* < 0.001, compared with the PBS-treated control by Dunnett’s multiple comparison test.

To verify the effectiveness of dosing and to measure the internal dose of Cd after treatment, we analyzed liver and kidney Cd concentrations. Cd concentrations in liver and kidney samples increased in a dose-dependent manner (Figure 1). Among all treatment groups, the highest concentrations were found in liver compared with kidneys (about two to three times higher in liver than in kidneys). The Cd concentrations in liver were between 58 and 2,833 ng/g wet weight, whereas in kidneys the Cd concentrations from 36 to 1,109 ng/g wet weight. EE_2_-treated and PBS control animals showed very low Cd concentrations both in liver and in kidneys.

### Luciferase activity

**Figure 2:**
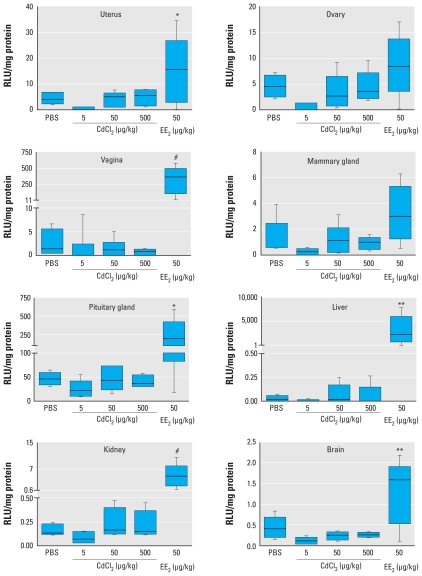
Tissue-specific expression of luciferase activity in uterus, ovary, vagina, mammary gland, pituitary gland, liver, kidney, and brain after SC exposure to CdCl_2_, EE_2_, or PBS for 3 consecutive days. Lines inside boxes represent median, boxes indicate 25th and 75th percentiles, and whiskers represent range. *p < 0.05, **p < 0.01, and ^#^*p* < 0.001 compared with the PBS-treated control by Dunnett’s multiple comparison test.

We measured luciferase activity in reproductive and nonreproductive organs to detect ER-mediated ERE-regulated gene expression (Figure 2). After EE_2_ exposure, uterus, vagina, pituitary gland, liver, kidney, and brain showed significantly increased luciferase activities compared with PBS controls (negative control). No significant alterations in luciferase activity were detected in any of the CdCl_2_-treated groups; however, nonsignificant decreases in luciferase activity were observed in several organs after exposure to the lowest dose of CdCl_2_ (5 μg/kg BW). Data on lung, heart, and WATs are not reported because luciferase activity levels in these tissues were not distinguishable from background in most of the vehicle- and CdCl_2_-treated animals. One of the EE_2_-treated animals showed very low levels of luciferase activity, which indicates a nonfunctional reporter. However, because the genotype of this mouse was confirmed, it was included in the statistical analysis.

### Western blot analysis of pErk, pAkt, and pMdm2 in liver

**Figure 3:**
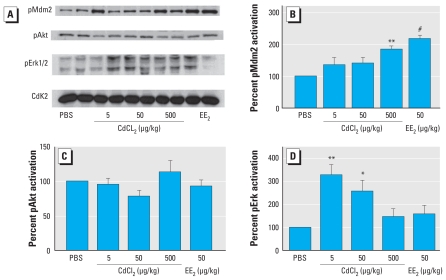
Western blots and densitometric analysis of phosphorylated (p) Erk, Akt, and Mdm2 in liver homogenates after SC exposure to CdCl_2_, EE_2_, or PBS for 3 consecutive days. (*A*) Representative Western blots. Densitometric analysis of Mdm2(Ser166) (*B*), Akt(Ser473) (*C*), and Erk(Tyr204) (*D*). Data were normalized against Cdk2, and the signal for the PBS vehicle control was set to 100%. Results represent mean ± SD. *p < 0.05, **p < 0.01, and ^#^*p* < 0.001, compared with PBS-treated controls by Dunnett’s multiple comparison test.

Western blot analysis of liver homogenates was performed to gain knowledge on the involvement of intracellular signaling pathways. Data showed significantly increased phosphorylation of Mdm2 at Ser166 in the high-dose CdCl_2_-treated (500 μg/kg BW) and EE_2_-treated animals, whereas no changes in phosphorylation of Akt at Ser473 was observed in CdCl_2_- or EE_2_-treated groups. Phosphorylation of Erk at Tyr204 was significantly increased after exposure to 5 or 50 μg of CdCl_2_/kg BW compared with the PBS control (Figure 3).

### Uterine wet weight and histology

**Figure 4:**
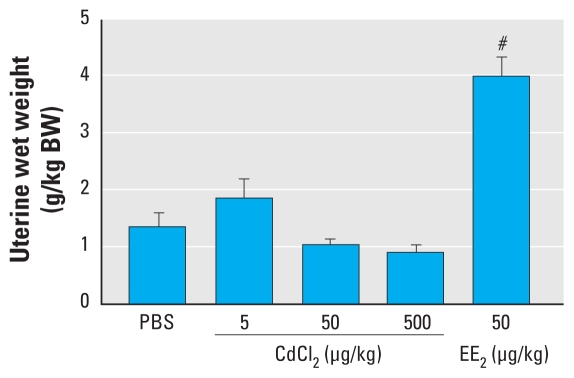
Body weight–adjusted uterine wet weights after SC exposure to CdCl_2_, EE_2_, or PBS for 3 consecutive days. Data shown are mean ± SD. ^#^*p* < 0.001 compared with PBS-treated controls by Dunnett’s multiple comparison test.

**Figure 5:**
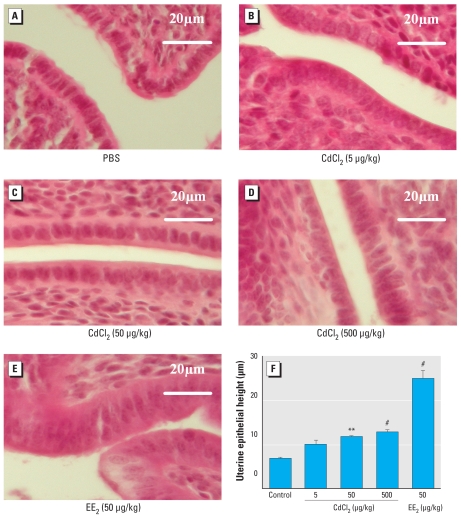
Height of the uterine luminal epithelium (μm) after SC exposure to CdCl_2_, EE_2_, or PBS for 3 consecutive days. (*A–E*) Representative photomicrographs of uterine tissue sections stained with hematoxylin and eosin (40×). (*F*) Quantitative evaluation of the uterine epithelium height (micrometers). Results represent mean ± S.D. **p < 0.01, and ^#^*p* < 0.001 compared with the PBS-treated control by Dunnett’s multiple comparison test.

Results of the uterotrophic assay showed that uterine wet weight was significantly increased after EE_2_ treatment (Figure 4). However, there was no increase in uterine wet weight in the CdCl_2_-treated animals, except that two of six animals in the low-dose group showed numerically higher (2.71 and 3.07 g/kg BW) uterine wet weights compared with PBS controls (mean, 1.53g/kg BW). Histological evaluation of uterine tissues showed that the height of the luminal epithelium was significantly increased in a dose-dependent manner after CdCl_2_-treatment. EE_2_ treatment also caused a significant increase in the height of uterine epithelium compared with PBS controls (Figure 5A–F).

## Discussion

In this study we investigated the estrogenicity of Cd in transgenic ERE-luciferase reporter mice by applying the OECD uterotrophic bioassay protocol. We obtained only limited evidence for estrogen-like effects by Cd—an increase in the height of the uterine luminal epithelium and changes in the phosphorylation of Erk1/2 and Mdm2 in the liver. Surprisingly, we detected no induction of the classical genomic ERα signaling pathway after Cd exposure, based on the lack of uterine growth and the lack of induction of the ERE-regulated luciferase reporter gene.

The luminal epithelial height and uterine wet weight are well-established end points for evaluating estrogenicity of chemicals (Branham et al. 1993, Diel et al. 2002; OECD 2007). In our examination of the histology of the uteri, we found a dose-dependent increase in the height of the luminal epithelium, which was almost doubled in the animals treated with 500 μg CdCl_2_/kg BW and four times higher in the EE_2_-treated mice compared with PBS controls; this is consistent with previous studies in rats (Höfer et al. 2009; Johnson et al. 2003; Zhang et al. 2007). We found no increase in uterine wet weight in animals treated with 50 or 500 μg CdCl_2_/kg BW; however, there were indications of an increase in uterine wet weight after treatment with 5 μg CdCl_2_/kg BW. In that group, two of six animals had uterine wet weights similar to those of the EE_2_-treated mice. Similar partial responses in CdCl_2_-treated animals have been recorded earlier in rats (Höfer et al. 2009). Our findings on uterine wet weight response after acute CdCl_2_ exposure support those of Zhang et al. (2007) and Höfer et al. (2009) at the doses investigated, but are in contradiction to those previously reported by Johnson et al. (2003). However, considering the differences in animal model and the experimental conditions used in previous studies, the dose range of Cd that could induce uterine weight increase is still under debate. Previously, Johnson et al. (2003) reported that uterine wet weight increased 1.7 times when ovariectomized rats were administered a single intraperitoneal (IP) injection of 5 μg CdCl_2_/kg BW. In another study using ovariectomized rats, the uterine weight increased after IP injection of 1,200 μg Cd^2+^/kg BW for 3 days, whereas no increase was observed with 120 μg Cd^2+^/kg BW (Zhang et al. 2007). More recently, Höfer et al. (2009) reported that uterine wet weight increased after a single IP injection of 500 or 2,000 μg CdCl_2_/kg BW; relative to controls, wet weight increased 1.2 and 1.4 times, respectively. However, no response was observed at lower doses.

Estrogen-induced responses in the uterus are mediated both by classical ERE-dependent and nonclassical ERE-independent ER signaling. Studies using gene-modified mice suggest that direct binding of ERα to DNA (i.e., classical estrogen signaling) is required for the uterine growth response (Ahlbory-Dieker et al. 2009; O’Brien et al. 2006), whereas the uterine luminal epithelium can respond to estrogen treatment even in the absence of classical signaling (O’Brien et al. 2006). We found that CdCl_2_ treatment did not affect uterine weight in the treated animals, nor did it induce the ERE-regulated reporter gene. These observations suggest that Cd might not activate classical estrogen signaling in the mouse uterus. However, CdCl_2_ treatment dose-dependently increased the height of the luminal epithelium. This suggests that Cd still possesses some estrogen-like activity in the uterus. Because the data imply an absence of classical signaling, nonclassical estrogen signaling should be considered as an alternative mechanism. In nonclassical signaling, estrogens promote ERα tethering to other transcription factors such as AP-1, Sp1, and nuclear factor-κB. Furthermore, estrogens can trigger rapid, nongenomic, membrane-associated effects. These types of estrogen activities cannot be detected in our mouse model in terms of reporter gene activation. Interestingly, recent *in vitro* work suggested that Cd can promote nonclassical transcriptional activation of ERα *in vitro* (Siewit et al. 2010). Furthermore, Cd promotes phosphorylation of Erk1/2, a marker of membrane-associated estrogen signaling, in the rat uterus *in vivo* (Zhang et al. 2007). It remains to be elucidated whether Cd can trigger ERα tethering or membrane-associated signaling in the mouse uterus and whether this activity would lead to the thickening of the luminal epithelium.

We investigated the effects of CdCl_2_ on classical ER signaling by analyzing ERE-regulated luciferase expression. As expected, our data showed significant increases in luciferase activity after SC exposure to the positive control EE_2_. However, we did not find any significant changes after SC injection of up to 500 μg CdCl_2_/kg BW. Instead of an increase, there was a nonsignificant decrease in the luciferase activity in most of the organs at relatively low doses of CdCl_2_ (5 μg/kg BW), which could reflect a weak antagonistic response. This observation is in line with a recently reported down- regulation of *C3* mRNA expression (an indicator of estrogenic activity) after IP injection of a single dose of 0.05–50 μg CdCl_2_/kg BW in rats (Höfer et al. 2009). Furthermore, regarding interaction of Cd with ER, Le Guevel et al. (2000) hypothesized that this interaction can occur only after the ERα has been activated by an agonist and that this interaction prevents ERα from binding to DNA and subsequently inhibits its transcriptional activity. This hypothesis was also supported by findings reported by Silva et al. (2006). Our findings on the ERE-regulated luciferase expression in response to CdCl_2_ exposure suggest a similar, antagonistic behavior of Cd interaction with ER in an *in vivo* setting.

Rapid phosphorylation of Erk1/2 MAPK in *in vitro* cell systems has been reported as an indicator of membrane-associated ER signaling (Liu et al 2008; Santen et al. 2002; Zang et al. 2009; Zivadinovic and Watson 2005). Activation of Erk1/2 is a widely used end point to reflect E_2_-induced nongenomic effects in a variety of *in vitro* cell systems (Filardo et al. 2000; 2002; Pedram et al. 2006; Razandi et al. 2003a, 2003b, 2004). Cd has been shown to activate Erk in MCF-7 cells *in vitro* and in rat uterus *in vivo* (Liu et al. 2008; Zhang et al. 2007); however, not all studies support these observations (Silva et al. 2006). Prompted by reports on the ERE-independent estrogen activity of Cd (Siewit et al. 2010: Zhang et al. 2007) and by our observations of the estrogen-like ERE-independent activity of Cd in the uterus, we explored the possible involvement of nonclassical ER signaling in Cd-mediated responses in our mouse model by analyzing pErk, pAkt, and pMdm2 contents in liver homogenates. We found a significant inversely dose-dependent activation of ERK1/2 after CdCl_2_ exposure. Compared with PBS controls, Erk phosphorylation was 3.5 times higher in animals exposed to 5 μg CdCl_2_/kg BW and 2.5 times higher in those exposed to 50 μg CdCl_2_/kg BW, but it was unchanged in response to EE_2_. This finding, together with the nonresponse in luciferase activity, suggests that Cd-induced phosphorylation of Erk is not mediated by classical ER signaling. Akt is considered a counterpart of Erk1/2 that could prevent cells from undergoing apoptosis induced by certain chemical or physical agents. However, we did not find any change in the amount of pAkt in either CdCl_2_- or EE_2_-treated groups.

Mdm2 is a negative regulator of the tumor suppressor p53, thus blocking p53 transcriptional activity and targeting p53 for proteasome-mediated degradation. Mdm2 interacts with various cellular proteins and is over-expressed in many human malignancies (Zang and Wang 2000). ER signaling has been shown to activate Mdm2, and Mdm2 is involved in the regulation of ERα turnover (Duong et al. 2007). We observed an activation of Mdm2 after CdCl_2_ (dose dependent) and EE_2_ treatment, indicating that Cd-induced estrogen-like responses may be mediated via nonclassical ER signaling and may contribute to promoting malignancies. Mdm2 phosphorylation induced by other environmental toxicants leads to attenuation of p53 response (Pääjärvi et al. 2005). However, further studies are required to clarify any role of Mdm2 phosphorylation in Cd induced toxicity.

Cd induces estrogenic responses differently in different studies, which may be because Cd uptake is strongly influenced by the route of exposure (Elsenhans et al. 1994; Höfer et al. 2009). In the present study, as expected, Cd contents in liver and kidney increased dose dependently after SC exposure to CdCl_2_, which reflects the effectiveness of dosing via SC injection. Internal dose in our study was similar to that reported by Zheng et al. (1996) after SC injection and by Höfer et al. (2009) after IP injection. Höfer et al. (2009) reported that Cd concentrations in uterus were similar to those in liver. In contrast, the observed Cd-mediated estrogen-like responses are different between studies. For instance, Höfer et al. (2009) reported that uterine wet weight and luminal epithelial height did not respond to oral administration of 0.5 and 4mg CdCl_2_/kg BW, but the same dosing regimen increased uterine *C3* mRNA expression 54 and 276 times, respectively. With regard to the route of exposure, Johnson et al. (2003) reported that *C3* mRNA expression was 12 times higher in rats after IP injection of 5 μg CdCl_2_/kg BW, whereas Höfer et al. (2009) observed a clear decrease in *C3* mRNA expression in rats with the same dose and route of exposure. This opposite response by Cd with the same dose and route of exposure in rats puts forward some new questions on its mode of action. Factors other than dose and mode of exposure, such as species/strain sensitivity, timing of exposure, age, and basal diet are likely to contribute to the net effects seen in the different studies.

## Conclusions

The present study shows that in the immature mouse model, Cd may induce a limited spectrum of estrogen-like effects in the uterus and liver without showing stimulation of ERE-mediated activation of ERα signaling (i.e., uterine weight gain or induction of ERE-regulated luciferase activity). Our findings suggest the involvement of other intracellular signaling pathways (i.e., regulation of non-ERE target genes; activation of membrane-associated ERα, G-protein coupled receptors, or growth factors, or cross-talk among them) in the biological activity of Cd. Further studies are needed to understand the molecular mechanisms involved in the estrogen-like activity of Cd and to clarify the conflicting data obtained in different *in vivo* and *in vitro* models used to investigate the estrogenicity of Cd. Our study provides some support for the association between Cd and the risk of hormone-related cancer observed in epidemiological studies (Åkesson et al. 2008; McElroy et al. 2006). Because all people have a lifelong exposure to Cd via food (Järup and Åkesson 2009; Satarug et al. 2010), this finding is of public health concern.

## Figures and Tables

**Table 1:** Body, liver, and kidney weights (mean ± SD) after SC exposure to CdCl_2_, EE_2_, or PBS for 3 consecutive days.

		Body weight (g)			Organ weight (g/kg BW)	
Treatment	*n*	Day 18	Day 22	Percent increase	Liver	Kidney
PBS control	5	8.44 ± 1.0	10.12 ± 0.91	16.6	50.8 ± 1.8	14.7 ± 0.6
CdCl_2_ (5 μg/kg)	6	8.58 ± 0.4	10.32 ± 0.38	16.8	50.4 ± 1.9	14.8 ± 1.0
CdCl_2_ (50 μg/kg)	6	8.61 ± 0.5	10.08 ± 0.62	14.6	49.3 ± 6.2	14.8 ± 0.7
CdCl_2_ (500 μg/kg)	5	8.66 ± 0.4	10.26 ± 0.44	15.6	51.6 ± 1.2	14.6 ± 0.7
EE_2_ (50 μg/kg)	5	8.50 ± 0.9	10.22 ± 0.99	16.8	56.9 ± 2.1*	14.6 ± 1.1

* *p* < 0.05 compared with the PBS control group by Dunnett’s multiple comparison test.
